# IP3 Receptor Type 2 Deficiency Is Associated with a Secretory Defect in the Pancreatic Acinar Cell and an Accumulation of Zymogen Granules

**DOI:** 10.1371/journal.pone.0048465

**Published:** 2012-11-21

**Authors:** Abrahim I. Orabi, Yuhuan Luo, Mahwish U. Ahmad, Ahsan U. Shah, Zahir Mannan, Dong Wang, Sheharyar Sarwar, Kamaldeen A. Muili, Christine Shugrue, Thomas R. Kolodecik, Vijay P. Singh, Mark E. Lowe, Edwin Thrower, Ju Chen, Sohail Z. Husain

**Affiliations:** 1 Department of Pediatrics, University of Pittsburgh Medical Center, Pittsburgh, Pennsylvania, United States of America; 2 Department of Internal Medicine, Yale University School of Medicine, New Haven, Connecticut, United States of America; 3 Department of Internal Medicine, University of Pittsburgh Medical Center, Pittsburgh, Pennsylvania, United States of America; 4 Department of Molecular Pathology, University of California San Diego, San Diego, California, United States of America; Technische Universität München, Germany

## Abstract

Acute pancreatitis is a painful, life-threatening disorder of the pancreas whose etiology is often multi-factorial. It is of great importance to understand the interplay between factors that predispose patients to develop the disease. One such factor is an excessive elevation in pancreatic acinar cell Ca^2+^. These aberrant Ca^2+^ elevations are triggered by release of Ca^2+^ from apical Ca^2+^ pools that are gated by the inositol 1,4,5-trisphosphate receptor (IP3R) types 2 and 3. In this study, we examined the role of IP3R type 2 (IP3R2) using mice deficient in this Ca^2+^ release channel (IP3R2^−/−^). Using live acinar cell Ca^2+^ imaging we found that loss of IP3R2 reduced the amplitude of the apical Ca^2+^ signal and caused a delay in its initiation. This was associated with a reduction in carbachol-stimulated amylase release and an accumulation of zymogen granules (ZGs). Specifically, there was a 2-fold increase in the number of ZGs (P<0.05) and an expansion of the ZG pool area within the cell. There was also a 1.6- and 2.6-fold increase in cellular amylase and trypsinogen, respectively. However, the mice did not have evidence of pancreatic injury at baseline, other than an elevated serum amylase level. Further, pancreatitis outcomes using a mild caerulein hyperstimulation model were similar between IP3R2^−/−^ and wild type mice. In summary, IP3R2 modulates apical acinar cell Ca^2+^ signals and pancreatic enzyme secretion. IP3R-deficient acinar cells accumulate ZGs, but the mice do not succumb to pancreatic damage or worse pancreatitis outcomes.

## Introduction

Pancreatitis is a painful, life-threatening disorder of the pancreas that results from numerous insults [1]. These include gallstones, alcohol abuse, trauma, medications, and metabolic disturbances. However, it is unclear why only a minority of individuals exposed to these noxious stimuli ever go on to develop pancreatitis. It is becoming increasingly evident that multiple environmental or genetic factors can predispose the host to disease [2], [3]. For example, most of the known gene mutations linked to pancreatitis, such as the serine protease inhibitor Kazal-type 1 (SPINK1) and cystic fibrosis transmembrane conductance regulator (CFTR), increase the likelihood of developing the disease, but do not appear to initiate it by themselves [4]. Exceptions are the gain of function mutations in the cationic trypsinogen gene, which cause acute or chronic pancreatitis with high penetrance [5].

The mechanisms underlying a predisposition to pancreatitis also appear to be multi-factorial. The pancreatic acinar cell is the main parenchymal cell of the exocrine pancreas [6]. Its primary function is to synthesize, store, and then secrete, upon stimulation, relatively large amounts of pancreatic digestive enzymes. Most of the enzymes are inactive precursors, or zymogens. They are densely packaged into zymogen granules (ZGs) that are localized to an apical region which faces the lumen. Physiologically, ZGs undergo massive compound exocytosis in response to Ca^2+^-activating secretagogues [7], [8]. The released zymogens travel through the pancreatic duct and become activated in the intestinal lumen by enterokinase at the brush border.

In the current study, we investigated the role of the apically concentrated intracellular Ca^2+^ channel the type 2 inositol 1,4,5-trisphosphate receptor (IP3R2) in coupling apical Ca^2+^ signals to ZG homeostasis. Further, we examined whether ZG accumulation in this setting would worsen pancreatitis. When acinar cells from mice with a global deletion of this particular Ca2+ channel (IP3R2^−/−^) [9] were stimulated with maximal concentrations of carbachol (1 uM), there were differences in apical Ca^2+^ signaling between IP3R2^−/−^ and wild type mice (WT). This was associated with a marked accumulation of ZGs. However, when mice were subjected to caerulein hyperstimulation, there were no differences in pancreatitis outcomes between IP3R2^−/−^ and WT.

**Figure 1:**
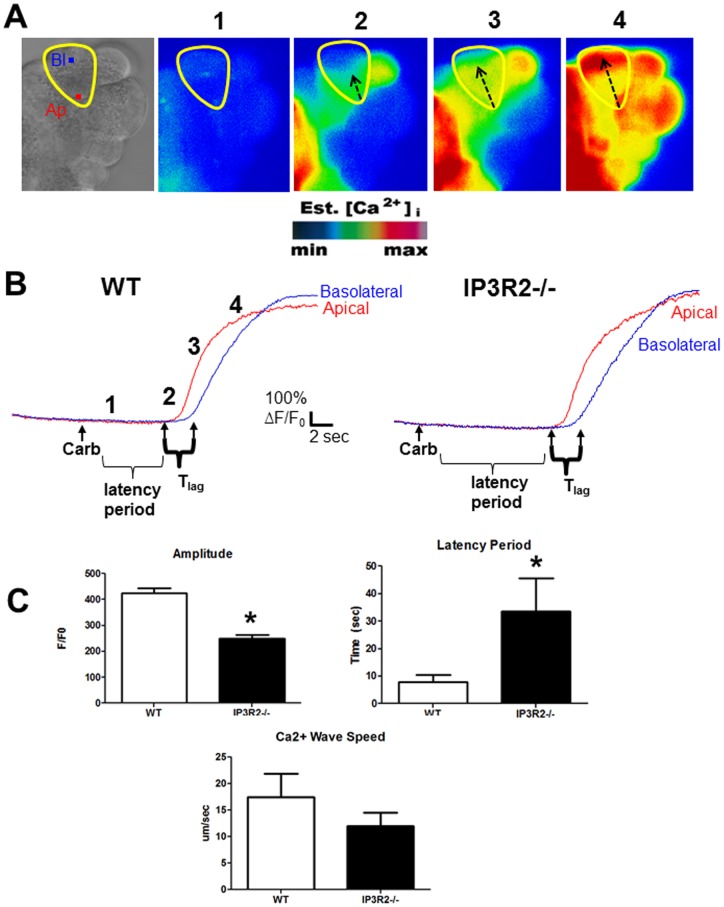
IP3R2-deficiency leads to reduced apical Ca^2+^ signals. (A) From left to right: Bright field view of an acinus, with a single acinar cell labeled at the apical (Ap) and basolateral (Bl) regions of interest. Fluorescent images of acini loaded with the Ca^2+^ indicator fluo-4 at baseline (1), shortly after stimulation with 1 uM carbachol (2), and subsequent images showing propagation of the Ca^2+^ wave from the apical to the basolateral region (3,4). (B) Each paneled image (1–4) also corresponds to a frame along a representative tracing of change in fluorescence over time for each region of interest. (C) Comparisons of the amplitude, latency period, and Ca^2+^ wave speed between WT and IP3R2^−/−^ cells (n = 25–40 cells in each group). The time from the administration of carbachol to the first Ca^2+^ rise in the apical region is the latency period. *, P<0.05 relative to WT.

## Methods

### Reagents and animals

All reagents were purchased from Sigma-Aldrich (St. Louis, MO) unless otherwise stated. IP3R2-deficient mice were generated on a Swiss Black background by Chen and colleagues, as previously described [9]. IP3R2 is completely ablated in the KO mice. They live to adulthood and do not have gross phenotypic changes. WT control mice were age-, sex- (male), weight- (20–25 gm), and strain-matched (Harlan Laboratories, Boston, MA). All of the animals were fed a standard laboratory chow, given free access to water, and randomly assigned to control or experimental groups. All experimental procedures and euthanasia were approved by the Institutional Animal Care and Use Committee (IACUC).

**Figure 2:**
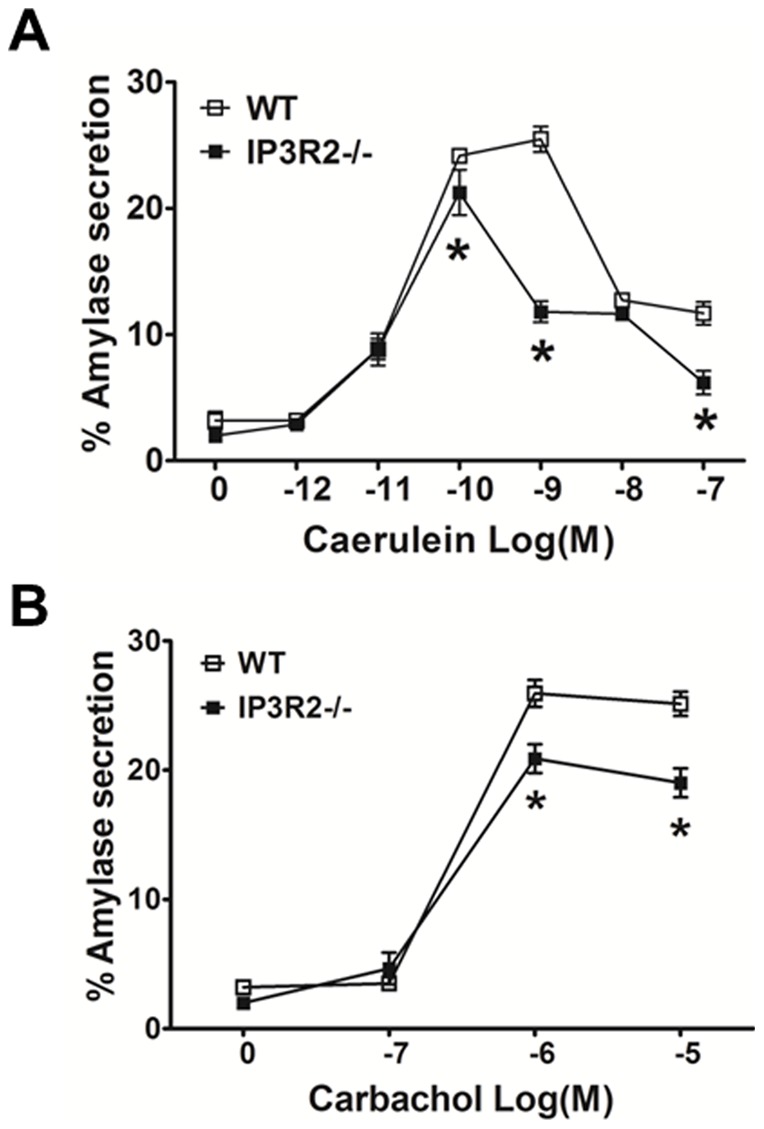
IP3R2-deficient acinar cells have reduced amylase secretion in response to Ca^2+^-activating agonists. Isolated acinar cells received (A) caerulein or (B) carbachol over a range of concentrations. Secretion was assayed after 30 min. (n = 3).*, P<0.05 relative to WT cells administered the same concentration of agonist.

### Detection of cellular Ca^2+^ signals

Groups of pancreatic acinar cells were isolated as previously described [10], with minor modifications. Briefly, the pancreas was removed, and then minced for 5 min in buffer containing 20 mM HEPES (pH 7.4), 95 mM NaCl, 4.7 mM KCl, 0.6 mM MgCl_2_, 1.3 mM CaCl_2_, 10 mM glucose, and 2 mM glutamine, plus 1% BSA, 1X MEM nonessential amino acids (Gibco Invitrogen, Carlsbad CA), 200 units/ml type-4 collagenase (Worthington, Freehold, NJ), and 1 mg/ml soybean trypsin inhibitor. The tissue was incubated for 30 min at 37°C with shaking at 90 rpm. The digest was transferred to a 15 mL conical tube and washed with collagenase-free buffer. The suspension was vigorously shaken for 15–20 seconds to separate the cells into smaller clusters. Acinar cells were loaded with the high-affinity Ca^2+^-sensing dye fluo-4/AM (K_Ca_ = 345 nM; Molecular Probes) as previously described [11]. Acinar cells were plated on acid-washed glass coverslips and then mounted on a perifusion chamber. Thereupon, they were stimulated with the muscarinic agonist carbachol (1 uM). A Zeiss LSM710 laser scanning confocal microscope was used with a 63X, 1.4 numerical aperture objective. The dye was excited at 488 nm wavelength, and emission signals of >515 nm were collected at frame speeds of 60–80 msec/frame. Fluorescence from individual acinar cells as well as apical and basal sub-cellular regions was recorded.

**Figure 3:**
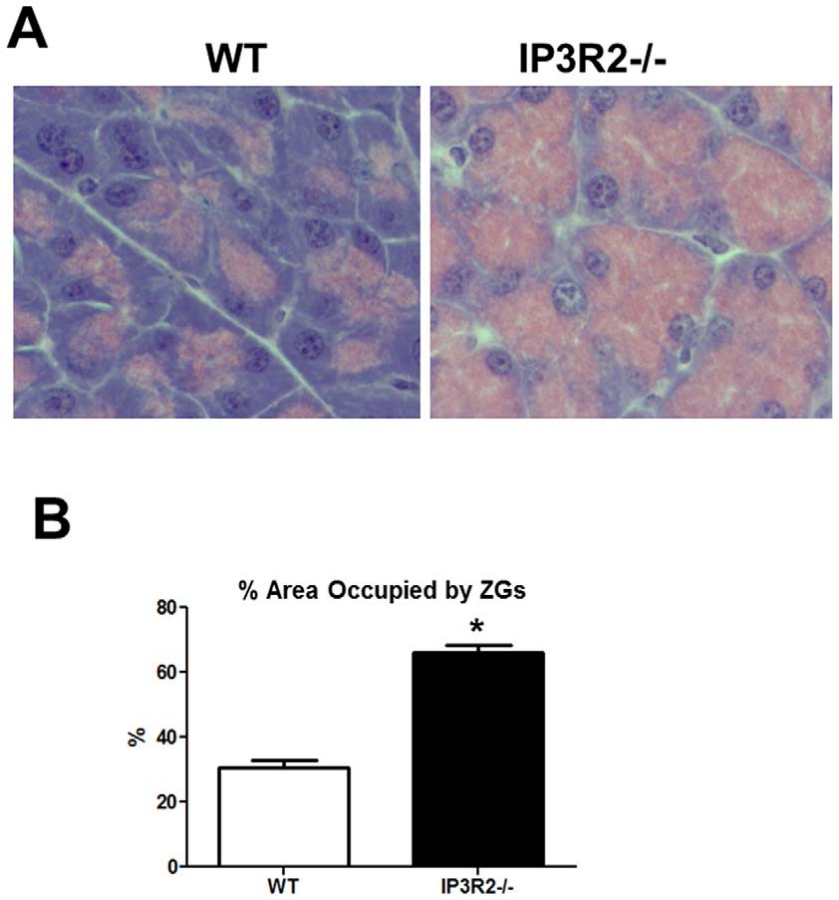
IP3R2-deficient mice have an increase in the pancreatic acinar cell ZG pool. (A) Hematoxylin and eosin staining of pancreas tissue from WT and IP3R2^−/−^ mice. (B) The area of the ZG pool is represented as percent of total cell area (n = 25–40 cells per condition). *, P<0.05 relative to WT.

**Figure 4:**
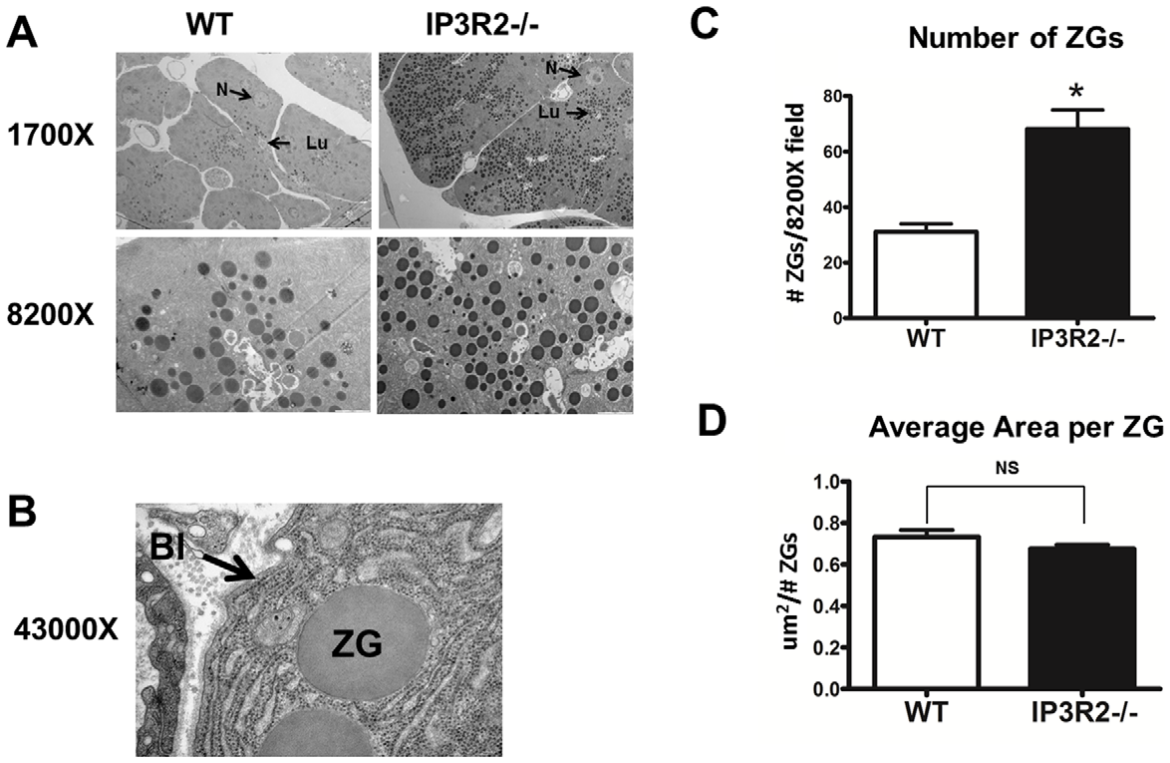
The pancreatic acinar cells of IP3R2-deficient mice contain a greater number of ZGs. (A) By EM, there was an increase in the number of ZGs. The nucleus (N) and lumen (Lu) of one cell from each lower power magnification is labeled (top row). (B) In some cells, ZGs could be seen extending into the basolateral (Bl) region. (C) Quantification of ZG number averaged over 20 fields at 8200X magnification. Each field contained one apical lumen that was positioned in the center of the field of view. Three to five cells converged at each lumen on cross section. (D) The average size of each ZG was unchanged between the two groups. *, P<0.05 relative to WT.

### Determination of Ca^2+^ wave speed, latency period, and amplitude

For Ca^2+^ wave speed analysis, apical and basal regions of interest in the acinar cell recordings were chosen using the ImageJ software (NIH, Bethesda, MD), and mean fluorescence over time in each region was graphed. Ca^2+^ wave speed was calculated by dividing the distance between the midpoints of the apical and basal regions of interest by the time it took for the Ca^2+^ wave to travel from the apical to the basal region. Latency period was defined as the time it takes to initiate a Ca^2+^ signal after administration of carbachol. The parameter does not vary with the size or position of individual acinar cells on a slide. Ca^2+^ amplitude was determined from apical regions of interest.

**Figure 5:**
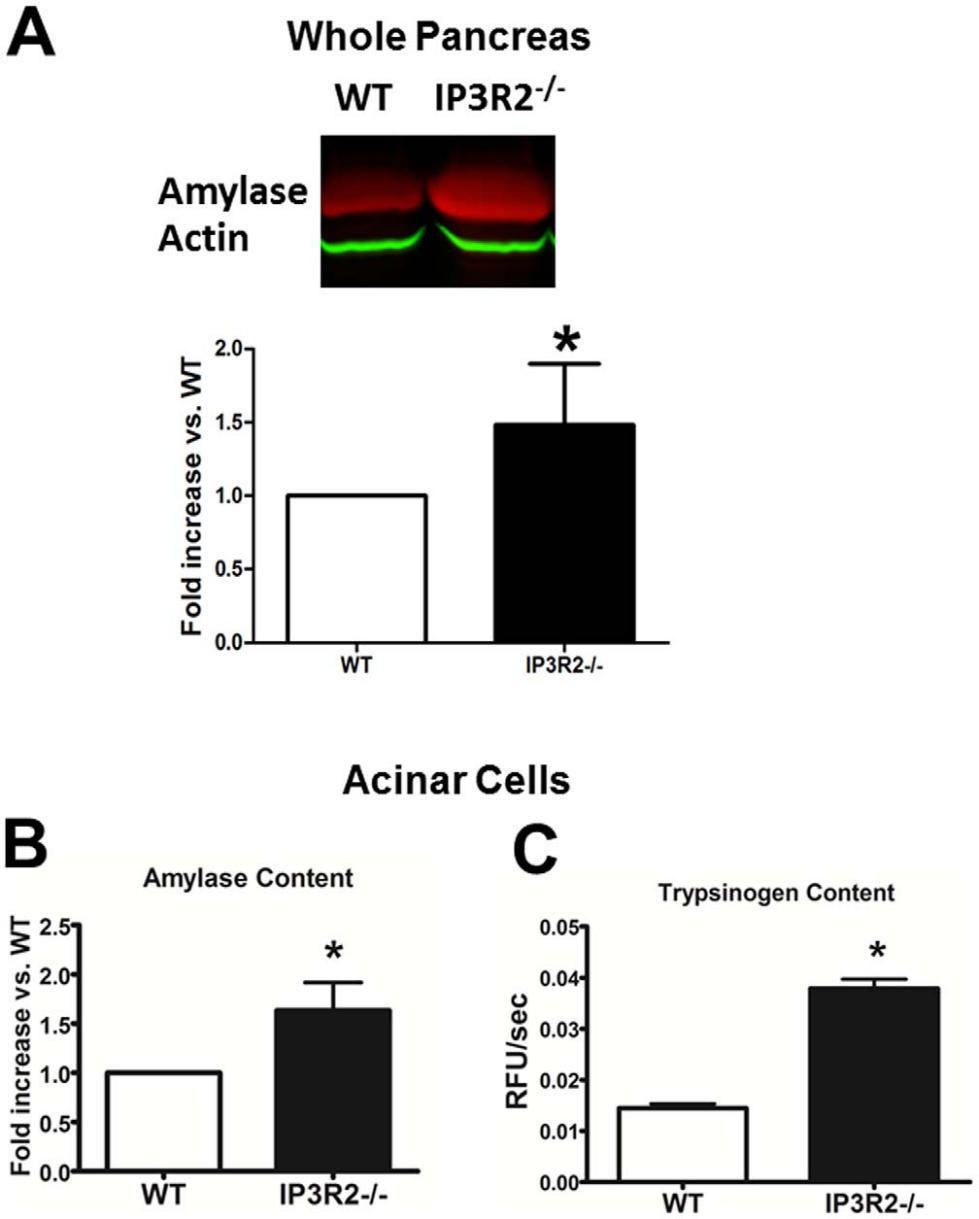
IP3R2-deficient mice express a greater amount of pancreatic enzyme. (A) Western blots were performed on pancreatic homogenates from WT and IP3R2^−/−^ mice for amylase and actin. Densitometry was expressed as fold increase over WT, normalized to actin (n = 3). (B) Amylase and (C) trypsinogen content were obtained by measuring the activities of each enzyme from lysed acinar cell suspensions of equal volume. Trypsinogen was activated to trypsin by incubating with enterokinase (1 uM) for 15 min prior to activity measurements (n = 3). *, P<0.05 relative to WT.

### Amylase secretion assays

For enzyme secretion studies, groups of pancreatic acinar cells were isolated as previously described [11], with modifications. Briefly, the pancreas was removed from euthanized mice and minced for 5 min in Dulbecco’s Modified Eagle Medium (DMEM)/F12 1X buffer without phenol red (Gibco Invitrogen, Carlsbad CA) plus 0.1% BSA, 2 mg/ml type-4 collagenase (Worthington, Freehold, NJ). The tissue was briefly oxygenated, and then incubated for 5 min at 37°C with shaking (90 rpm). The buffer was exchanged with fresh collagenase, then briefly oxygenated, and incubated for 35 min. The tissue digest was filtered through a 300 um mesh (Sefar American, Depew, NY) to yield acinar cells, which were then washed three times with a collagenase-free buffer and allowed to equilibrate for 5 min at 37°C before treatment. Amylase was measured using a Phadebas kit (Magle Life Sciences, Lund, Sweden). Secretion was calculated from cultured primary acinar cells by dividing amylase released into the media by total amylase content.

**Figure 6:**
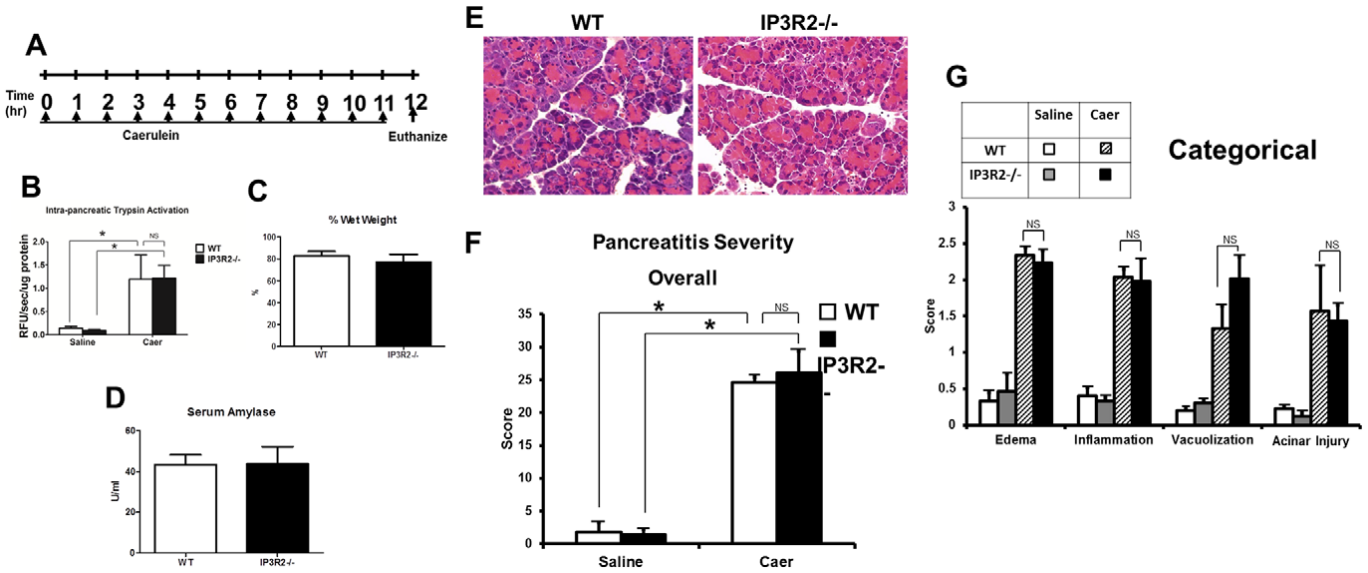
IP3R2-deficiency does not modulate the severity of caerulein-hyperstimulation pancreatitis. (A) Schema for pancreatitis induction with hourly intra-peritoneal injections of (Caer)ulein (50 ug/kg). Mice were euthanized 12 hr after the first caerulein injection, and serum amylase. (B) Intra-pancreatic trypsin activity, (C) percent wet weight of pancreas and (D) serum amylase from mice given caerulein were assayed. (E) Representative hematoxylin and eosin sections of the pancreas from caerulein treated mice, along with (F) overall and (G) categorical histological severity scores (n = 3 animals per group). *, P<0.05 relative to saline-treated controls.

**Figure 7:**
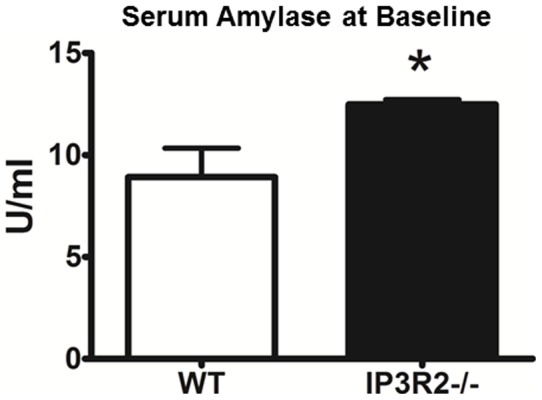
IP3R2-deficient mice have higher serum amylase levels at baseline. Serum amylase was assayed from WT and IP3R2^−/−^ mice (n = 3). *, P<0.05 relative to WT.

### Preparation of tissue for light and electron microscopy

For light microscopy, pancreatic tissue was fixed at room temperature for 2 hr in 10% formalin solution with 125 mM phosphate buffer (pH 7.4), then transferred to 70% ethanol. Paraffin-embedded sections cut at a thickness of 5 um were stained with hematoxylin and eosin and imaged on a light microscope. For electron microscopy (EM), animals were anesthetized and *in vivo* perfused for 5 min with 20 mL of fixative containing 2% paraformaldehyde and 2.5% glutaraldehyde in 0.1 M cacodylate buffer. The pancreas was dissected, cut into 2 mm^3^ tissue blocks, incubated at room temperature for 2 hr in the remaining fixative, and then post-fixed with 1% osmium tetroxide. Tissues were blocked, stained with 2% uranyl acetate, dehydrated in acetone series, and Epon embedded. Thin sections were stained with lead citrate and uranyl acetate. Electron micrographs were acquired on a Philips 410 electron microscope.

### Immunoblotting

Briefly, 25 mg of freshly isolated pancreas was snap frozen and stored at −80°C. Samples were then thawed and homogenized in 400 ul of buffer containing 1 mM ethylenediaminetetraacetic acid (EDTA), 10 mM Tris, 100 mM NaCl, and 1X protease inhibitor cocktail (Roche, Indianapolis, IN). Protein content was assayed following the method of Bradford [12], and 10 ug were loaded per lane on a 10% SDS gel. Proteins were transferred to a PVDF membrane and amylase was probed using a monoclonal antibody (ab20319; Abcam, Cambridge, MA). Bands were detected using IRDye 800CW goat anti-mouse and goat anti-rabbit secondary antibodies (Li-Cor, Lincoln, NE). Results were normalized to a mouse monoclonal antibody to β-actin (A2228), and represented as fold increase over WT.

### Trypsin activation and trypsinogen content

To measure trypsin activity from whole tissue, pancreas stored at −80°C was thawed and homogenized in iced medium containing 5 mM MOPS, 250 mM sucrose, and 1 mM MgSO4 (pH 7.0). Samples were then centrifuged at 1000 g for 5 min at 4°C. Active trypsin was measured from supernatant as previously described [13], using a fluorogenic substrate (Peptides International, Louisville, KY) which had the amino acid sequence Boc-Gln-Ala-Arg-MCA. Trypsinogen content was obtained by incubating cell lysates with enterokinase (1 uM) for 15 min prior to activity measurements.

### Caerulein hyperstimulation in vivo and histological grading

Pancreatitis was induced in mice by administering hourly intra-peritoneal injections of caerulein (50 ug/kg body weight) for 12 hr, as previously described [14]. Saline-injected animals served as controls. Paraffin-embedded sections were stained with hematoxylin and eosin and evaluated using a 20X objective over 10 separate fields in a blinded fashion. Pancreas tissue was graded for edema, acinar cell vacuole formation, inflammation, and acinar injury, as previously described [15], according to a 0–3 point scale. For serum amylase measurements, whole blood was centrifuged at 5000 g for 10 min at 4°C. Serum amylase was measured using a Phadebas kit (Magle Life Sciences, Lund, Sweden).

### Statistical analysis

Statistical significance was determined using a Student’s t-test. Data are expressed as mean ± SEM unless otherwise stated. A p-value of <0.05 was considered significant.

## Results

### Ca^2+^ signals in IP3R2^−/−^ acinar cells

We examined the Ca^2+^ signal in IP3R2^−/−^ acinar cells using a maximally stimulating concentration of the Ca^2+^-activating agonist carbachol (1 uM) [16], [17]. Consistent with previous reports [18], [19], we found that there was a reduction in the amplitude of the apical Ca^2+^ signal by 41%, compared to WT acinar cells (Fig. 1). In addition, there was a delay among the IP3R2^−/−^ acinar cells of 25.7 sec in the latency period, which is the time from the application of carbachol to the initiation of the Ca^2+^ signal (P<0.05). There were no statistical differences in the propagation of the Ca^2+^ wave from the apical to basal region when comparing IP3R2^−/−^ with WT mice.

### Secretagogue-stimulated amylase secretion is reduced in IP3R2^−/−^ acinar cells

Isolated acinar cells from WT and IP3R2^−/−^ mice were stimulated with a concentration range of caerulein or carbachol, and amylase secretion was measured after a 30 min incubation period (Fig. 2). Amylase secretion was reduced in the IP3R2^−/−^ acinar cells compared to WT primarily at the maximal secretory concentrations of caerulein (10^−10^ M) and carbachol (10^−6^ M).

### ZGs accumulate in IP3R2^−/−^ acinar cells

The pancreatic acinar cell is a highly polarized cell [20]. ZGs are localized to the apical pole, while the nucleus lies close to the basolateral region. By hematoxylin and eosin staining, ZGs in WT acinar cells can be seen as pink granules within the apex [21] (Fig. 3). This ZG pool within the acinar cells of IP3R2-deficient mice, however, appeared to increase and extend towards the nucleus and basolateral region. Estimates of the size of this pool were made by measuring the cross-sectional area of the ZG pool in relation to the area of acini. From these measurements, the total acinar cell area between IP3R2^−/−^ and WT was unchanged. However, the ZG pool occupied 66.2% of the acinar cell compared with 38.8% in the WT state (P<0.05). The increase was seen homogenously throughout the pancreas and in all of the knockout mice. To determine whether the increase in the pancreatic acinar cell ZG pool was due to a larger size of the ZGs or a greater number of ZGs, EM was performed at magnifications up to 43000X (Fig. 4). Consistent with the light microscopy findings, sections by EM from the IP3R2^−/−^ mice demonstrated an extension of ZGs in most cells up to the nucleus and, in some cases, close to the basolateral region. There was a 2-fold increase in ZG number (P<0.05), although the average area of each granule remained unchanged.

### ZG content is increased in IP3R2^−/−^ acinar cells compared to WT

To examine whether an observed increase in the number of ZGs translates to higher amounts of zymogens, western blotting was performed in whole pancreas (Fig. 5). Using equal protein loading, actin levels were not increased. On the other hand, amylase was increased by 49% (P<0.05). To confirm this, total amylase content was measured in equal volumes of lysed acinar cells using a substrate activity assay. Amylase content was increased by 50% in the IP3R2^−/−^ mice (P<0.05). This result is in contrast to the report by Mikoshiba and colleagues, who demonstrated that there was no basal increase in amylase by immunoblot [18]. The zymogen expressed in highest quantities by the acinar cell is trypsinogen [22]. Trypsinogen content was measured from acinar cell lysates by first cleaving the zymogen to its active form trypsin (in the presence of enterokinase) and then measuring trypsin activity using a specific fluorogenic substrate assay. As with amylase, trypsinogen content was also increased in the IP3R2^−/−^ acinar cells, but by 2.6-fold (P<0.05). The results demonstrate that IP3R2-deficient mice have a selective increase in pancreatic acinar cell ZG number and zymogen content.

### IP3R2-deficiency does not worsen caerulein pancreatitis severity

We next examined whether IP3R2^−/−^ mice would succumb to worse pancreatitis outcomes in a mild, interstitial model that is induced by repeated hourly injections of supraphysiologic concentrations of caerulein [23] (Fig. 6). At baseline, the IP3R2^−/−^ mice had no histological evidence of pancreatic acinar cell injury, edema, immune cell infiltration, or vacuolization. Twelve hours after caerulein hyperstimulation, intra-pancreatic trypsin activation, tissue wet-weight, serum amylase, and pancreatic histology were equally increased in both WT and IP3R2^−/−^ mice. Despite the absence of pancreatic damage at baseline, we found that serum amylase was higher at baseline in the IP3R2^−/−^ mice by 32%, compared to the WT (P<0.05; Fig. 7).

## Discussion

In this study, using IP3R2^−/−^ mice, we found that the IP3R2 contributes to apical Ca^2+^ signals, and its absence is associated with an accumulation of ZGs. Intracellular Ca^2+^ pools within the acinar cell are gated primarily by two major Ca^2+^ channels, the IP3R and the ryanodine receptor [10], [24], [25]. IP3Rs are classically triggered by a cascade that begins with ligand binding of G protein-coupled receptors, activation of phospholipase C, generation of inositol 1,4,5-trisphosphate (IP3) from phosphatidylinositol 4,5-bisphosphate, and IP3 binding to the IP3R [26]. There are three isoforms of the IP3R, but acinar cells predominantly express type 2 and 3, in roughly equal amounts [27], [28]. Both IP3R2 and IP3R3 are localized to the apical region. We observed a reduction in the amplitude of the apical Ca^2+^ signal in the IP3R2^−/−^ mice, which is consistent with previous reports [18], [29]. Interestingly we also found a prolongation of the latency period, that is, the time it took to initiate a Ca^2+^ signal after administration of carbachol. A similar increase in the latency period would be expected in cell types with a similar distribution of IP3Rs. Like acinar cells, hepatocytes contain a small, apical region, called the bile canaliculus, where IP3R2 is also concentrated [30]. Hepatocytes from IP3R2^−/−^ mice have reduced Ca^2+^ amplitude and a delayed rise time, which is another indication of slowed opening [31]. Although the authors did not measure the latency period in this study, in another paper, they showed that IP3R2 knockdown in primary cultured hepatocytes causes a prolonged Ca^2+^ latency period [32]. One explanation for the lag in the latency period is that the IP3R2^−/−^ acinar cells simply have fewer IP3Rs, since there is no compensatory increase in IP3R1 or IP3R3 [29]. This could result in fewer stochastic openings or less clustering of IP3Rs [33]. Another explanation is isoform specificity. In the absence of IP3R2, IP3R3 is the dominant isoform at the apical region of the acinar cell, where the Ca^2+^ signal begins. IP3R3 has a lower affinity for IP3 than does IP3R2 [34], which would mean that, in the absence of IP3R2, a greater amount of IP3 is necessary to initiate a Ca^2+^ signal. It’s also possible that IP3R isoforms have distinct subcellular localization in the acinar cell, even within the ER of the subapical region. This could also account for differences in opening kinetics [35]. Additional differences in the IP3R2 isoform include a greater sensitivity to ATP [29] and a negative modulatory effect on store-operated Ca^2+^ entry (SOCE) channels [36].

Acinar cell IP3Rs conduct spatially localized apical Ca^2+^ signals that are necessary for secretion of ZG contents into the lumen of the pancreatic duct [37]. We found that the IP3R2^−/−^ acinar cells had a partial reduction in amylase secretion at carbachol concentrations that were maximal, consistent with findings by Dr. Mikoshiba and colleagues in which they used an IP3R2^−/−^ mouse that they had generated [18]. By contrast, they found that the IP3R2/3 DKO mouse acinar cells had a complete secretory block and, *in vivo*, manifested signs of pancreatic insufficiency.

In both the IP3R2/3 DKO mouse as well as the IP3R2^−/−^ we used, the reduction in secretion was associated with an accumulation of ZGs. There were twice as many ZGs, seen extending beyond the apical region and abutting into the supranuclear and basolateral regions. However, neither the size of the ZGs nor the size of the acinar cells changed. Interestingly, trypsinogen content was greater in the IP3R2^−/−^ mouse acini than was amylase, implying that there was less release of trypsinogen than amylase. This raises the possibility of the selective release of ZG contents. There is evidence that ZGs vary in the composition of their cargo contents [38], either among different populations of acinar cells within regions of the gland (e.g. the peri-insular area [39]) or possibly even within individual cells. Upon stimulus-secretion coupling, there is also support for the differential release of contents [40]. Thus our results could indicate that IP3R-dependent mechanisms control the exocytosis of selective pools of ZGs that, for example, house relatively larger amounts of trypsinogen over amylase. Alternatively, there are distinct secretory pathways for certain proteins via constitutive, constitutive-like, or minor secretory pathways [41], that are independent of Ca^2+^ or IP3R opening [42].

IP3R2 deficiency and its associated accumulation of ZGs were insufficient to cause spontaneous pancreatic damage or to worsen pancreatitis severity. The reason why serum amylase was higher in the IP3R2^−/−^ mouse at baseline could be due to pancreatic enzyme exit from the cell through basolateral exocytosis, a phenomenon that is triggered by VAMP8 (vesicle-associated membrane protein 8)-mediated ZG-membrane fusion [43]. VAMP8^−/−^ mice have reduced zymogen secretion [43], [44], [45], due to a defect in compound exocytosis [8], and they also develop ZG accumulation [45]. But they are protected against pancreatitis [43], [45]. Similarly, the IP3R2/3 DKO mice, which also accumulate ZGs, are protected against ethanol-induced protease activation [46], [47]. In summary, IP3R2 modulates apical acinar cell Ca^2+^ signals and pancreatic enzyme secretion. IP3R-deficient acinar cells accumulate ZGs, but the mice do not succumb to pancreatic damage or worse pancreatitis outcomes.

## References

[1] Frossard J-L, Steer ML, Pastor CM (2008) Acute pancreatitis. The Lancet 371: 143–152. 10.1016/S0140-6736(08)60107-5 18191686

[2] Whitcomb DC (2006) Acute Pancreatitis. N Engl J Med 354: 2142–2150. 16707751 10.1056/NEJMcp054958

[3] Bai HX, Ma MH, Orabi AI, Park A, Latif SU, et al (2011) Novel characterization of drug-associated pancreatitis in children. J Pediatr Gastroenterol Nutr 53: 423–428. 21681111 10.1097/MPG.0b013e318228574e PMC3626448

[4] LaRusch J, Whitcomb DC (2011) Genetics of pancreatitis. Curr Opin Gastroenterol 27: 467–474. 21844754 10.1097/MOG.0b013e328349e2f8 PMC3704192

[5] Whitcomb DC, Gorry MC, Preston RA, Furey W, Sossenheimer MJ, et al (1996) Hereditary pancreatitis is caused by a mutation in the cationic trypsinogen gene. Nat Genet 14: 141–145. 8841182 10.1038/ng1096-141

[6] Pandol SJ, Saluja AK, Imrie CW, Banks PA (2007) Acute Pancreatitis: Bench to the Bedside. Gastroenterology 132: 1127–1151. 17383433 10.1053/j.gastro.2007.01.055

[7] Palade G (1975) Intracellular aspects of the process of protein synthesis. Science 189: 347–358. 1096303 10.1126/science.1096303

[8] Behrendorff N, Dolai S, Hong W, Gaisano HY, Thorn P (2011) Vesicle-associated membrane protein 8 (VAMP8) is a SNARE (soluble N-ethylmaleimide-sensitive factor attachment protein receptor) selectively required for sequential granule-to-granule fusion. J Biol Chem 286: 29627–29634. 21733851 10.1074/jbc.M111.265199 PMC3283193

[9] Li X, Zima AV, Sheikh F, Blatter LA, Chen J (2005) Endothelin-1-induced arrhythmogenic Ca2+ signaling is abolished in atrial myocytes of inositol-1,4,5-trisphosphate(IP3)-receptor type 2-deficient mice. Circ Res 96: 1274–1281. 15933266 10.1161/01.RES.0000172556.05576.4c

[10] Husain SZ, Prasad P, Grant WM, Kolodecik TR, Nathanson MH, et al (2005) The ryanodine receptor mediates early zymogen activation in pancreatitis. Proc Natl Acad Sci U S A 102: 14386–14391. 16186498 10.1073/pnas.0503215102 PMC1242288

[11] Orabi AI, Shah AU, Muili K, Luo Y, Mahmood SM, et al (2011) Ethanol enhances carbachol-induced protease activation and accelerates Ca2+ waves in isolated rat pancreatic acini. J Biol Chem 286: 14090–14097. 21372126 10.1074/jbc.M110.196832 PMC3077610

[12] Bradford MM (1976) A rapid and sensitive method for the quantitation of microgram quantities of protein utilizing the principle of protein-dye binding. Anal Biochem 72: 248–254. 942051 10.1016/0003-2697(76)90527-3

[13] Chaudhuri A, Kolodecik TR, Gorelick FS (2005) Effects of increased intracellular cAMP on carbachol-stimulated zymogen activation, secretion, and injury in the pancreatic acinar cell. Am J Physiol Gastrointest Liver Physiol 288: G235–243. 15458924 10.1152/ajpgi.00334.2004 PMC2975016

[14] Orabi AI, Shah AU, Ahmad MU, Choo-Wing R, Parness J, et al (2009) Dantrolene mitigates caerulein-induced pancreatitis in vivo in mice. Am J Physiol Gastrointest Liver Physiol 299: G196–204. 10.1152/ajpgi.00498.2009 PMC2904115 20448143

[15] Wildi S, Kleeff J, Mayerle J, Zimmermann A, Bottinger EP, et al (2007) Suppression of transforming growth factor {beta} signalling aborts caerulein induced pancreatitis and eliminates restricted stimulation at high caerulein concentrations. Gut 56: 685–692. 17135311 10.1136/gut.2006.105833 PMC1942167

[16] Owyang C, Logsdon CD (2004) New insights into neurohormonal regulation of pancreatic secretion. Gastroenterology 127: 957–969. 15362050 10.1053/j.gastro.2004.05.002

[17] Lugea A, Gong J, Nguyen J, Nieto J, French SW, et al (2010) Cholinergic mediation of alcohol-induced experimental pancreatitis. Alcohol Clin Exp Res 34: 1768–1781. 20626730 10.1111/j.1530-0277.2010.01264.x PMC2950903

[18] Futatsugi A, Nakamura T, Yamada MK, Ebisui E, Nakamura K, et al (2005) IP3 Receptor Types 2 and 3 Mediate Exocrine Secretion Underlying Energy Metabolism. Science 309: 2232–2234. 16195467 10.1126/science.1114110

[19] Park HS, Betzenhauser MJ, Won JH, Chen J, Yule DI (2008) The type-2 InsP3 receptor determines the sensitivity of InsP3-induced Ca2+ release to ATP in pancreatic acinar cells. J Biol Chem. 10.1074/jbc.M804184200 PMC2533790 18658132

[20] Petersen OH, Tepikin AV (2008) Polarized calcium signaling in exocrine gland cells. Annu Rev Physiol 70: 273–299. 17850212 10.1146/annurev.physiol.70.113006.100618

[21] Kern H (1993) Fine Structure of the Human Exocrine Pancreas. In: Go V, editor. The Pancreas: Biology, Pathobiology, and Disease. 2 ed. New York: Raven Press. 9–20.

[22] Sahin-Toth M, Toth M (2000) High-affinity Ca(2+) binding inhibits autoactivation of rat trypsinogen. Biochem Biophys Res Commun 275: 668–671. 10964720 10.1006/bbrc.2000.3355

[23] Niederau C, Ferrell LD, Grendell JH (1985) Caerulein-induced acute necrotizing pancreatitis in mice: protective effects of proglumide, benzotript, and secretin. Gastroenterology 88: 1192–1204. 2984080 10.1016/s0016-5085(85)80079-2

[24] Petersen OH (2005) Ca2+ signalling and Ca2+-activated ion channels in exocrine acinar cells. Cell Calcium 38: 171–200. 16107275 10.1016/j.ceca.2005.06.024

[25] Thorn P, Lawrie AM, Smith PM, Gallacher DV, Petersen OH (1993) Local and global cytosolic Ca2+ oscillations in exocrine cells evoked by agonists and inositol trisphosphate. Cell 74: 661–668. 8395347 10.1016/0092-8674(93)90513-p

[26] Foskett JK, White C, Cheung KH, Mak DO (2007) Inositol trisphosphate receptor Ca2+ release channels. Physiol Rev 87: 593–658. 17429043 10.1152/physrev.00035.2006 PMC2901638

[27] Wojcikiewicz RJ, Luo SG (1998) Differences among type I, II, and III inositol-1,4,5-trisphosphate receptors in ligand-binding affinity influence the sensitivity of calcium stores to inositol-1,4,5-trisphosphate. Mol Pharmacol 53: 656–662. 9547355 10.1124/mol.53.4.656

[28] Wojcikiewicz RJ (1995) Type I, II, and III inositol 1,4,5-trisphosphate receptors are unequally susceptible to down-regulation and are expressed in markedly different proportions in different cell types. J Biol Chem 270: 11678–11683. 7744807 10.1074/jbc.270.19.11678

[29] Park HS, Betzenhauser MJ, Won JH, Chen J, Yule DI (2008) The type 2 inositol (1,4,5)-trisphosphate (InsP3) receptor determines the sensitivity of InsP3-induced Ca2+ release to ATP in pancreatic acinar cells. J Biol Chem 283: 26081–26088. 18658132 10.1074/jbc.M804184200 PMC2533790

[30] Hirata K, Pusl T, O'Neill AF, Dranoff JA, Nathanson MH (2002) The type II inositol 1,4,5-trisphosphate receptor can trigger Ca2+ waves in rat hepatocytes. Gastroenterology 122: 1088–1100. 11910359 10.1053/gast.2002.32363

[31] Cruz LN, Guerra MT, Kruglov E, Mennone A, Garcia CR, et al (2010) Regulation of multidrug resistance-associated protein 2 by calcium signaling in mouse liver. Hepatology 52: 327–337. 20578149 10.1002/hep.23625 PMC3025771

[32] Hernandez E, Leite MF, Guerra MT, Kruglov EA, Bruna-Romero O, et al (2007) The Spatial Distribution of Inositol 1,4,5-Trisphosphate Receptor Isoforms Shapes Ca2+ Waves. Journal of Biological Chemistry 282: 10057–10067. 17284437 10.1074/jbc.M700746200 PMC2825872

[33] Rahman T (2012) Dynamic clustering of IP3 receptors by IP3. Biochem Soc Trans 40: 325–330. 22435806 10.1042/BST20110772

[34] Newton CL, Mignery GA, Sudhof TC (1994) Co-expression in vertebrate tissues and cell lines of multiple inositol 1,4,5-trisphosphate (InsP3) receptors with distinct affinities for InsP3. J Biol Chem 269: 28613–28619. 7961809

[35] Vermassen E, Parys JB, Mauger JP (2004) Subcellular distribution of the inositol 1,4,5-trisphosphate receptors: functional relevance and molecular determinants. Biol Cell 96: 3–17. 15093123 10.1016/j.biolcel.2003.11.004

[36] Lur G, Sherwood MW, Ebisui E, Haynes LP, Feske S, et al.. (2011) IP3 receptors and Orai channels in pancreatic acinar cells: co-localisation and its consequences. The Biochemical journal. 10.1042/BJ20110083 PMC3262233 21568942

[37] Kasai H, Augustine GJ (1990) Cytosolic Ca2+ gradients triggering unidirectional fluid secretion from exocrine pancreas. Nature 348: 735–738. 1701852 10.1038/348735a0

[38] Mroz EA, Lechene C (1986) Pancreatic zymogen granules differ markedly in protein composition. Science 232: 871–873. 2422756 10.1126/science.2422756

[39] Malaisse-Lagae F, Ravazzola M, Robberecht P, Vandermeers A, Malaisse WJ, et al (1975) Exocrine pancreas: evidence for topographic partition of secretory function. Science 190: 795–797. 1105788 10.1126/science.1105788

[40] de Dios I, Garcia-Montero AC, Orfao A, Manso MA (1999) Selective exocytosis of zymogen granules induces non-parallel secretion in short-term cholecystokinin-stimulated rats. J Endocrinol 163: 199–206. 10556768 10.1677/joe.0.1630199

[41] Castle AM, Huang AY, Castle JD (2002) The minor regulated pathway, a rapid component of salivary secretion, may provide docking/fusion sites for granule exocytosis at the apical surface of acinar cells. J Cell Sci 115: 2963–2973. 12082156 10.1242/jcs.115.14.2963

[42] Chaudhuri A, Husain SZ, Kolodecik TR, Grant WM, Gorelick FS (2007) Cyclic AMP-dependent protein kinase and Epac mediate cyclic AMP responses in pancreatic acini. Am J Physiol Gastrointest Liver Physiol 292: G1403–1410. 17234888 10.1152/ajpgi.00478.2005 PMC2975017

[43] Cosen-Binker LI, Binker MG, Wang CC, Hong W, Gaisano HY (2008) VAMP8 is the v-SNARE that mediates basolateral exocytosis in a mouse model of alcoholic pancreatitis. J Clin Invest 118: 2535–2551. 18535671 10.1172/JCI34672 PMC2413188

[44] Wang CC, Shi H, Guo K, Ng CP, Li J, et al (2007) VAMP8/endobrevin as a general vesicular SNARE for regulated exocytosis of the exocrine system. Mol Biol Cell 18: 1056–1063. 17215514 10.1091/mbc.E06-10-0974 PMC1805086

[45] Wang CC, Ng CP, Lu L, Atlashkin V, Zhang W, et al (2004) A role of VAMP8/endobrevin in regulated exocytosis of pancreatic acinar cells. Dev Cell 7: 359–371. 15363411 10.1016/j.devcel.2004.08.002

[46] Gerasimenko JV, Lur G, Ferdek P, Sherwood MW, Ebisui E, et al (2011) Calmodulin protects against alcohol-induced pancreatic trypsinogen activation elicited via Ca2+ release through IP3 receptors. Proc Natl Acad Sci U S A 108: 5873–5878. 21436055 10.1073/pnas.1016534108 PMC3078340

[47] Gerasimenko JV, Lur G, Sherwood MW, Ebisui E, Tepikin AV, et al (2009) Pancreatic protease activation by alcohol metabolite depends on Ca2+ release via acid store IP3 receptors. Proc Natl Acad Sci U S A 106: 10758–10763. 19528657 10.1073/pnas.0904818106 PMC2696551

